# Everyday executive function issues from the perspectives of autistic adolescents and their parents: Theoretical and empirical implications

**DOI:** 10.1177/13623613231224093

**Published:** 2024-01-19

**Authors:** Lorcan Kenny, Anna Remington, Elizabeth Pellicano

**Affiliations:** 1 University College London, UK; 2 Macquarie University, Australia

**Keywords:** attention, monotropism, phenomenology

## Abstract

There is a long tradition of research into autistic people’s executive function skills. Yet, despite decades of research on EF in autism, the existing literature remains contradictory and ‘confusing’, with a large – and unresolved – discrepancy between small-to-moderate effect sizes demonstrated on laboratory-based EF tasks and large effect sizes on questionnaire-based everyday EF measures. We sought to understand this mismatch between ‘lab and life’ by inviting 12 autistic adolescents (12–19 years) and their mothers (*n* = 7) to convey their views and perspectives about their own, or their child’s, EF skills as they transition to adulthood. We followed Braun and Clarke’s method for reflexive thematic analysis using an inductive approach. Participants told us that their EF skills were highly variable, acutely dependent on the context in which they were deployed and potentially related to differences in the way that they process information more broadly. Participants’ reports provided rare insights into their and their children’s executive control – insights that do not straightforwardly map onto traditional theoretical models of EF. Future work on EF needs to take seriously the perspectives and subjectivity of autistic people themselves, including by triangulating quantitative, objective assessments with qualitative, subjective reports in complementary (controlled, uncontrolled) settings.

**Lay abstract:**

Autism researchers have a long-time interest in a set of skills called executive function. These skills include planning, inhibition, and switching between one activity and another. There was a theory that these skills explained the social and thinking difficulties autistic people might have. After years of study into this, the evidence is confusing and contradictory. Autistic people tend to report struggling quite a lot with these skills. Yet, when researchers test these skills, they do not tend to find such big difficulties. In this study, we spoke to 12 autistic teenagers and seven of their mothers about this. We asked them what they thought about their own, or their child’s, executive function skills. We wanted to know about things they were good at and things they struggled with. They told us that their skills were very changeable from one context to the next and from one time to the next. According to their reports, their skills depended on how motivated they were by doing the task. Another thing that influenced their skills were how anxious they felt at the time they needed to use the skill. Finally, they told us that sometimes they think differently about how best to perform a task. We discuss what these insights mean for autism researchers who study these skills. In future, research should ask people about their experiences alongside testing their abilities in different contexts. Combining these information sources will give us a better understanding of autistic people’s everyday skills as well as how best to support them.

It has long been held that autistic people have problems in executive function (EF) (Damasio & Maurer, 1978; Ozonoff et al., 1991; Russell, 1997) – those high-level cognitive skills, including planning, cognitive shifting, working memory and inhibition, necessary for flexible, goal-oriented behaviour (Norman & Shallice, 1986; Zelazo & Müller, 2011). Yet, despite decades of research, the literature on autistic people’s EF remains ‘confusing’ (Kenworthy et al., 2008), demonstrating group differences of only moderate effect (Demetriou et al., 2018). This is despite EF challenges regularly being reported in questionnaire measures of real-world EF (e.g. Kenworthy et al., 2005; R. A. Lawson et al., 2015), and regularly described by autistic people (Cribb et al., 2019; DePape & Lindsay, 2016; Livingston et al., 2019; Pellicano et al., 2021).

One potential reason for this is the weak ecological validity of traditional, lab-based EF measures (Burgess et al., 2006). Research has tended to study EF within carefully controlled lab-based settings, relying heavily on structured behavioural paradigms designed for the computer or table-top. There are good reasons for this methodological approach. It allows researchers to attempt to simplify and control the experimental situation, focusing on the executive processes of interest and reducing the influence of extraneous factors, such as variation in non-executive processes and measurement error. Nonetheless, the almost exclusive reliance on lab-based research means that performance on EF tasks often does not translate to the more complex situations that people experience in everyday life (Broadbent, 1991; Burgess et al., 2006; Manchester et al., 2004). Indeed, traditional, lab-based EF measures have been charged with lacking in both *representativeness* – the correspondence between the task and real-life settings, and *generalisability* – the degree to which task performance predicts problems in real-life settings (Burgess et al., 2006; Franzen & Wilhelm, 1996; Kvavilashvili & Ellis, 2004).

This issue – the mismatch between ‘lab and life’ – may be further exacerbated for autistic people. Some have suggested that autistic people perceive the world in a fundamentally different way because they rely more on incoming sensory input and less on prior knowledge (Friston et al., 2013; R. P. Lawson et al., 2014; Pellicano & Burr, 2012). On this view, perceptual and cognitive differences should emerge particularly when levels of uncertainty are high. As a result, autistic people might be less likely to show difficulties on tasks when tested in relatively controlled, decontextualised circumstances; rather, differences should occur predominantly when uncertainty mounts, particularly in less controlled, real-world contexts. Research on autistic EF might therefore be disproportionately disadvantaged by its overreliance on controlled, laboratory research – which could lead to fundamental misunderstandings of the nature of executive challenges for autistic people.

How, then, should we go about trying to understand the real-life executive skills of autistic people? Some researchers have proposed that it is not possible to understand the complexities of cognitive processes like EF based solely on ‘objective’ observations of people’s behaviour (Jack & Roepstorff, 2002; see also Kingstone et al., 2008). Instead, they suggest we need to adopt a multi-layered approach, which triangulates the more objective (‘third person’) components of the conventional scientific method – systematic observation and measurement (Kingstone et al., 2008) – with qualitative (‘first-person’ perspective) methods (Jack & Roepstorff, 2002). Yet, such subjective, first-person experiences are usually eschewed by experimental psychologists, for whom ‘experience is still regarded as a problem, rather than a resource ready to be tapped’ (Jack & Roepstorff, 2002, p. 334).

This problem of trusting the usefulness of first-person experiences is intensified in the case of autism research. Some researchers have suggested that autistic people lack the ability for self-reflection (Lombardo et al., 2011), thus questioning the veracity of autistic people’s accounts of their own experiences (Frith & Happé, 1999; Zahavi, 2010). Consequently, researchers have often avoided turning to first-person autistic testimony, preferring to privilege reports from informants (e.g. parents and teachers) or lab-based observation over considering the perspectives of the person themselves (Mazefsky et al., 2011; see Milton, 2012, for discussion). Yet, research has demonstrated that autistic people can have a deep capacity to reflect on many aspects of the self, regardless of their intellect or communication preferences (Baggs, 2005; McGeer, 2004; Nicolaidis et al., 2015). Indeed, autistic young people have been shown to demonstrate great awareness of their EF challenges when asked directly using questionnaire-based measure (Kenworthy et al., 2022) and indirectly using interview-based measures (Browning et al., 2009; Cribb et al., 2019).

Existing qualitative studies have revealed some information about autistic people’s executive function issues, particularly with regard to ‘compensatory’ strategies (Livingston et al., 2019), and challenges with planning and organisation, and flexibility, in the contexts of home (e.g. Cribb et al., 2019; Heyworth et al., 2023; Ludlow et al., 2012), school (e.g. Carrington & Graham, 2001; Humphrey & Lewis, 2008) and work (e.g. DePape & Lindsay, 2016). All of these studies, however, have elicited such information *indirectly*, having identified such issues in their conduct of studies on autistic people’s broader experiences. There are, to our knowledge, no existing studies that have *directly* elicited the subjective, qualitative experiences of autistic people. Here, we sought to address this gap by asking autistic young people specifically about their EF skills, thereby implementing the first step of Jack and Roepstorff’s (2002) multi-layered approach to understanding everyday cognition: eliciting subjective phenomenological experiences of the construct of interest. Our aims were to elicit autistic young people’s everyday executive experiences and to determine whether such experiences map on to the construct as it is understood in the scholarly literature and typically assessed in the laboratory.

We also interviewed young people’s parents (see also Kenworthy et al., 2022). Critically, we did not seek convergence between children and parents’ reports. Instead, we approached the issue of everyday executive function from both perspectives to provide a richly complex understanding of the topic, in the spirit of Richardson and St. Pierre’s (2005) concept of ‘crystallisation’. We focused on adolescence because it is a time in which EF skills are undergoing significant development (Blakemore & Choudhury, 2006; Diamond, 2014; Uddin, 2021) and the external demands on these skills increase as young people embark on the transition to adulthood. Specifically, we asked: what are the views and perspectives of autistic young people and their parents about their own, or their child’s, EF skills as they make the transition to adulthood?

## Method

### Participants

Nineteen participants were recruited through community contacts in London and surrounding areas to participate in this study, including 12 autistic adolescents without an intellectual disability (11 male, one female), aged between 12 and 19 years, and seven of their parents (all mothers; no fathers volunteered to take part). One mother spoke about her two autistic children and one mother participated whose child decided not to be involved in the study. Specific data on mothers’ background, including their socioeconomic status, racial/ethnic background and autistic identity, were not recorded.

All adolescents had received an independent clinical diagnosis of an autism spectrum condition, as reported by parents, and all showed clinically-significant autistic features (*t*-score of at least 60) on the parent-reported, Social Responsiveness Scale, second edition (SRS-2; Constantino & Gruber, 2012). Some participants also had one or more co-occurring conditions (Table 1). All but one was in education at the time of interview, although the nature of the setting varied.

**Table 1.** Background information of our young person interviewees.

Participant ID	Sex	Age	Co-occurring conditions^a^	IQ classification^b^	School setting
001	Female	16	DCD, PDA	Low average	Not in education
002	Male	12	None	High average	Mainstream^c^
003	Male	13	ADHD, DCD, Tourette’s	High average	Special^d^
004	Male	14	None	Average	Mainstream^e^
005	Male	18	None	Borderline	Mainstream^f^
006	Male	14	None	Low average	Mainstream^f^
007	Male	19	ADHD, DCD, Dyslexia	Average	FE college
008	Male	14	None	Average	Mainstream
009	Male	13	ADHD	Average	Mainstream^c^
010	Male	12	None	Average	Home educated
011	Male	14	None	High average	Mainstream^f^
012	Male	15	None	Low average	Mainstream^f^

*Note.* IQ = intellectual quotient; DCD = developmental coordination disorder; PDA = pathological demand avoidance; ADHD = attention deficit hyperactivity disorder; FE = further education.

a As reported by parents.

b IQ classification = classification based on the full-scale intellectual quotient (IQ) from the Wechsler Abbreviated Scale of Intelligence, second edition (WASI-II; Wechsler, 2011); Borderline = 71 < IQ < 80, Low average = 81 < IQ < 90, Average = 91 < IQ < 110, High average = 111 < IQ < 120, Superior = 121 < IQ < 130, Very superior = 131 < IQ < 140.

c Mainstream school with a support base that is not autism-specific.

d An autism-specific special school.

e A mainstream school with an autism-specific support base.

f A mainstream classroom with a teaching assistant.

### Measures

#### Semi-structured interviews

We conducted individual, semi-structured interviews, during which we asked participants to reflect on their own or their child’s EF abilities. We began by asking participants a general question about how they or their child were currently getting on, with respect to school and home life. Next, we adopted a critical incident technique (Flanagan, 1954), whereby participants were asked to recollect specific situations where they or their child either (a) had excelled at, or (b) had difficulties employing EF abilities. Our questions focused on higher-order abilities, such as the ability to manage their time, to multitask, to retain information and to adapt flexibly to changes in task demands. We used the same interview schedule with all participants, although questions were reworded to suit their different roles. Questions were sufficiently broad to allow participants to introduce specific experiences and to elaborate on themes important to them; probe questions were asked when necessary (See supplementary materials).

Interviews were recorded with participants’ prior permission and transcribed verbatim. One young participant opted not to have their interview recorded and so detailed notes were taken by the interviewer.

### Data analysis

We used reflexive thematic analysis (Braun & Clarke, 2019) to analyse participants’ responses. We adopted an inductive (bottom-up) approach where we identified themes within a ‘contextualist’ framework of critical realism (Bhaskar, 1978). Critical realism lies between a realist ontology, which asserts that only one objective state of reality exists, irrespective of the researcher’s or participant’s views on it (Jenkins, 2010) and a relativist ontology, which asserts that reality is entirely constructed through historical, political and social interchanges (Schwandt, 1994). It acknowledges that we all have subjective experiences (the empirical), that an objective reality exists outside of our experience (the actual) and that causal mechanisms lie between and within these domains (the real) (Fletcher, 2017). Critical realism is suited to this study because it allows for EF difficulties existing because of brain-based differences, irrespective of participants’ views on them (the actual reality) but also allows space to tap into participants’ subjective experiences of these difficulties (the empirical reality) and facilitates discussion of how these might interact (the real reality).

Our analysis was informed by our training in psychology, our positionalities as non-autistic researchers, and by our broad alliance with the neurodiversity paradigm (see Pellicano & den Houting, 2022; Walker, 2014). One experienced researcher (EP) immersed herself in the data, reading all transcripts twice, taking reflexive notes and applying codes to each transcript (managed in NVivo, version 12). Data were initially coded separately by informant (autistic young people, mothers). It soon became apparent, however, that many of the codes and potential themes were common across informants. All transcripts were therefore combined and coded as one data set, and re-coded where necessary. Codes were clustered together to identify candidate themes and subthemes during multiple discussions with LK, during which we were alert to points of convergence *and* divergence between young people and parents’ perspectives, which we highlight in the analysis below. EP then generated a draft thematic map, and the relevant data were collated under each theme and subtheme before being reviewed again by all authors. Analysis was therefore iterative and reflexive (Braun & Clarke, 2019).

### Procedure

Ethical approval for this study was granted through UCL Institute of Education. All participants provided written informed consent prior to participation. Participants completed individual semi-structured interviews face-to-face in a quiet room at the university (*n* = 8) or over the phone (*n* = 11), according to participant preference.

### Community involvement statement

Community members were not involved in any aspect of the research process.

## Results

**Figure 1.**
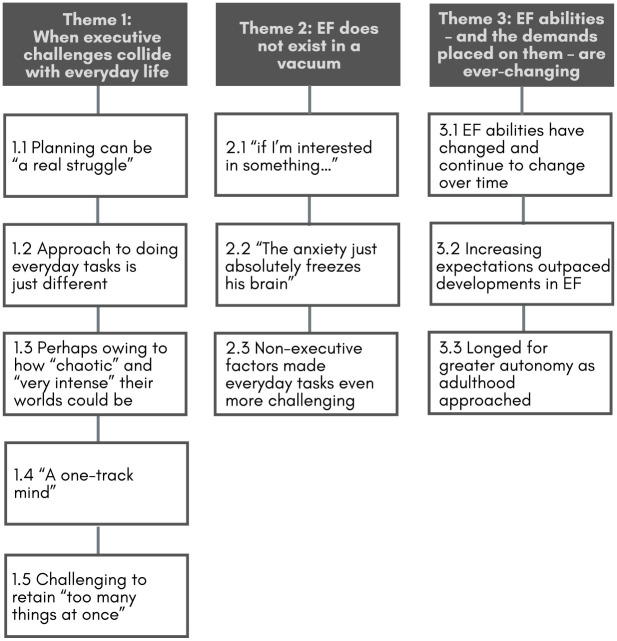
Autistic young people and their parents’ experiences of everyday executive challenges: themes and subthemes.

We identified three themes in the data (Figure 1). Below, themes are highlighted in bold, subthemes are italicised, and quotes are attributed via participant ID numbers (P= parent; YP = young person).

### Theme 1: when executive challenges collide with everyday life

Autistic young people and their mothers gave rich descriptions of the way they or their children process information and navigate day-to-day activities across a range of contexts. They highlighted that *planning can be* ‘*a real struggle*’ (subtheme 1.1) (P305). Many young people acknowledged that ‘I can’t keep myself organised, I’m just like that’ (YP1516). Another said, while ‘I do things lastminute.com^1^ [laughs] . . .being disorganised like that can be a real burden to me’ (YP1116). Their mothers agreed, with some suggesting that planning and organisational difficulties were ‘the most difficult thing he has actually . . .that is the one that I’m most concerned about as a mother for his future’ (P305). This same mother worried that ‘it’s not only the fact that he’s not organised; it’s that he’s actually not conscious about it’. Another mother agreed:

he was planning to go away on a school trip. He will come home with a list of items, but he would never think, ‘Maybe, I need to pack these’. Or ‘I need to take these’. Or ‘I need to start packing’. (P1118)

One mother also noted how her child, when asked his lunch preferences, would always respond with ‘I’ll just have the same as yesterday’. She explained: ‘there’s almost an element of it’s easier to do that than to think and organise anything different, for risk it might go wrong or just because it’s hard’ (P1508).

These planning differences caused parents to reflect on how their children achieved ‘internalised processing of routine’ – the ‘getting dressed, cleaning teeth, washing your hands or flushing the toilet, I don’t know how and why he chooses which things he remembers and doesn’t remember, and how to do all of that’ (P1508). Nevertheless, mothers reported doing much of the day-to-day organising for their children, to a much greater extent than their other (non-autistic) children: ‘on his free days, I make a schedule and I let him tick it off – then he does it and he can be pretty structured with that, but if I don’t do this, he wastes his time’ (P1116).

Planning ahead and organising were not uniformly challenging, however. Some young people reported that they ‘do think ahead sometimes’ (YP524): ‘I don’t write it out, but I do organise myself roughly in my head’ (YP1508). Mothers also reported instances in which their children were ‘good at preparing and knowing when to start getting ready and that type of thing . . .like meeting friends on time, planning his journey, knowing when he needs to get on the train’ (P506). They also noted, however, that they felt their children’s *different ways of approaching everyday tasks* (subtheme 1.2) was not always intuitive or as ‘efficient’ as they could be and relied on prior knowledge that their children hadn’t acquired:

I started giving him the job of emptying the dishwasher . . . I realised he was taking each individual knife or fork, walking with one piece of cutlery, putting it in the drawer and then wandering back again with the next one. I said, ‘You can pick up the whole cutlery basket, put it over by the drawer, and then, it’s really easy – look, all the knives, all the forks’. It was just giving him that idea, this is how to do it – but, obviously, that depends on me actually seeing him doing the job. (P524)

One autistic young person contested these views, however, describing how he just organises himself in a different way:

When people say something’s not organised, what they don’t normally understand is that it’s organised for the person who organised it. I understand where everything should be in my mind . . .it’s not that I’m unorganised, just that some people think I’m unorganised because of the system I use. (YP1508)

Autistic young people and their mothers felt that their own or their children’s challenges were at least in part owing to *how* ‘*chaotic*’ *and* ‘*very intense*’ *their worlds could be* (subtheme 1.3) – both in terms of their own inner lives and the realities of an often-unpredictable world. Parents described how their children’s brains were ‘ticking all the time . . .it just, like, keeps going and never stops’ (P1129) and ‘there’s always something going over and over and over in his mind . . .he has told us that his mind is always whirring’ (P1506). Having to deal with ‘lots of things at once’ (YP1506) often meant young people could struggle ‘to sift out what’s important versus what’s not important’ (P1508).

Young people often dealt with this challenge by ‘procrastinating a lot, saying I will do something and then doing it later and having to do it all in one sitting – then I’ll be very stressed out’ (YP1529). Parents agreed and suggested that their children’s tendency to procrastinate was ‘because he doesn’t know where to start’ (P1508). This mother went on to explain that her child ‘does enjoy getting things out of the way, so the procrastination is not because he doesn’t want to do it; it’s just that he can’t get started’ (P1508). When speaking of plans being changed, young people agreed: ‘if someone changes the plan, I tend to get stuck on the original plan . . .it’s because I can’t normally think of anything else’ (YP524). Sometimes, these challenges were such that they found ‘it ridiculously hard to get out of bed in the morning’ (YP1116).

Our interviewees also attributed some of the ways in which they navigated their everyday lives to *having* ‘*a one-track mind*’ (subtheme 1.4) – ‘it’s like my mind, like subconsciously or consciously, just prioritises one piece of information over the other, and for that reason I’m not able to remember everything’ (YP1116). Mothers reported how: ‘if I’ve told him something and then before he’s finished doing that, dad has told him to do something different, he’s like, ‘Which do you want me to do? I can’t do both’’ (P1508). Young people agreed: ‘if I’m doing something first, I can’t do a second one at the same time. I just find it difficult’ (YP1118).

Not being able ‘to do more than one thing at a time’ (YP1529) had its consequences, however. It often meant that they had ‘to finish a project or whatever I’m doing before I can start something else . . .it would affect me, if I couldn’t’ (YP1129). Parents also emphasised how ‘having to see it through once he starts something’ (P1118) can be debilitating:

if she’s doing something that she really likes, she’s like, ‘leave me alone for now and then I’ll finish and we can go’. And I’ll say, ‘ok – I’ll come back when you’re done’. Because she will get upset – she can’t just leave it. She has to finish it. (P1129)

One young person said:

It can sometimes be quite complicated to focus on more than one thing at a time . . .I suppose it’s in the same way that a computer can’t handle multi-tasking. I would prefer to process and solve a problem one at a time rather than using strategies that other, neurotypical people would use to solve problems. So, in that sense, I guess multi-tasking I’m not necessarily good at because if I have to do more than one thing at once, that can sometimes be quite stressful. (YP1506)

This serial style of processing could also make it difficult to generate alternative task plans, especially when the circumstances change, and the goals might not be personally meaningful:

Say if I said to him, ‘You need to get 100 grams of milk chocolate’, if they didn’t have milk chocolate, he would just not get anything at all, whereas my other son might think, ‘oh well, I’ll get dark chocolate’. It would have to be what was on the list, otherwise he wouldn’t get it. (P506)

Sometimes, being so focused on a single task led to ‘being engrossed in things’ (P1129), which participants described as a real strength: ‘he’s very good at focusing . . .amazing actually, what he can remember and what he can do’ (P506). The same absorption, however, could also make it difficult for children to engage in everyday activities: ‘His mind is whirring so much about all the stuff he’s researched and wants to research, that he doesn’t focus on trivial things like [domestic chores]’ (P1506). This mother also noted that, in these moments, her child is difficult to engage: ‘we’re constantly badgering him . . .but it’s like he hasn’t heard because he is so focused on whatever he is doing in his mind’ (P1506). Other parents agreed that ‘he tends to get very absorbed in one thing, rather than being able to do a few things at the same time’ (P506) to the extent that ‘it’s like he’s zoned out of the real world and he can’t listen to you’ (P1508). This can mean that ‘it can take days to do one piece of homework’ (YP1508), they ‘can lose track of time’ (YP1529), and can be difficult to ‘disengage’ (P1508): ‘if there’s one activity that I’m interested in, then when we move onto the next activity my concentration on that activity can be affected because the activity before I’m still thinking about in my head’ (YP1506). This same participant felt that this attentional focus was distinctively related to being autistic: ‘my neurotypical friends are better at channelling and organising the energy they put into each subject [at school] and try and balance that’. Another young person recounted some negative side effects:

Sometimes I do completely forget to take breaks, and often the times when I do eventually go on the break, I completely lose the flow of where I was going with things. And in those circumstances, it can actually be quite disastrous to go on a break. (YP1116)

Many participants also reported that they or their children find it *challenging to retain* ‘*too many things at once*’ (subtheme 1.5), especially ‘if it’s quite a complicated set of instructions’ (P506). They had difficulty remembering ‘list of instructions or directions – those things you just need to hold for short amounts of time’ (P1508). Young people agreed: ‘even if I pay attention, if it’s something that slips my mind, it just pops out’ (YP1516). Parents reported their children often asking ‘Oh, what do I have to do next?’ (P1129). Mothers were often perplexed by these difficulties, describing them as having ‘a photographic memory’ (P1129) or being ‘a complete sponge . . .I’ve no idea how he’s able to retain so much’ (P1506). These challenges dealing with ‘too many steps’ (YP1116) could occur across contexts, including at school ( ‘I know she [teacher] has explained it, but I can’t remember. When I come to do it, I can’t remember what it was she said to me that I had to do’; YP506), at ‘the shops’ (P1129), at work ( ‘my employers just give me one instruction, I complete that instruction and then they give me another one, because they know that I can’t work with three or four complete different instructions’; YP1116) and at home:

I always find that with instructions, if I say, ‘Can you put that washing in the basket and take that washing out of the machine’, then by the time he’s got downstairs, he’s like, ‘Where am I putting that washing again?’ So, it’s when you ask him more than one thing at a time, that seems to throw him. It might just be he processes the information in a different way, so he needs the information one bit at a time, in little chunks. (P524)

### Theme 2: EF does not exist in a vacuum

Despite describing many everyday EF challenges, participants were adamant that these challenges were not fixed; rather, they were critically affected by other factors. One key factor was their or their children’s degree of task motivation – that is, ‘*if I’m interested in something . . .*’ (subtheme 2.1). Young people clearly described how their EF skills can ‘depend on the scenario or situation. If it’s something in which I’m extremely focused on in terms of interest, if those factors are generally high, then I plan ahead of time quite well’ (YP1506).

Participants gave rich examples of how motivation affected their own or their children’s EF skills. One mother recounted how ‘it really depends on what he’s doing. If it’s his bricklaying, absolutely fine. If it’s instructions with something he doesn’t really want to do, like maths, he’s quite likely to forget. So, it’s what’s important to him, really’ (P506). Another recounted how her child could successfully plan when ‘things motivate him . . .but not everything motivates him. That’s the problem’ (P305).

Young people agreed that:

If it’s something which I really don’t like, I find that I’m often a lot slower than if it’s something which I really do like . . .And yeah, sometimes my Mum has said stuff like, ‘Once you’ve finished your college assignment, you can play your clarinet’ and I’m just like, ‘Well, seeing as I really don’t enjoy clarinet playing, I don’t think I’ll ever get [laughter] this assignment done’. (YP1116)

While motivation could cause participants to utilise their EF skills effectively, anxiety had the opposite effect. As one mother put it, ‘*the anxiety just absolutely freezes his brain*’ (subtheme 2.2). They described how their children can manage ‘until his anxiety goes completely through the roof and he feels kind of unwell or headache or stomach-ache and he’s feeling anxious – that has a massive impact on his life. Enormous’ (P1506). Others, too, reported how ‘sometimes when her mental state is in a good place, I think she might be able to manage slightly. But when it is not, then it is mayhem’ (P1129). Her daughter confirmed this view. When speaking of her inability to attend school, which the school interpreted as a sign of inflexibility, she said, ‘just I get built up in my head and that stopped me from physically actually being able to get there’ (YP1129). One mother felt that this anxiety was part and parcel of being autistic: ‘the autism definitely produces this huge anxiety all the time, and when you’re anxious, planning becomes much more painful’ (P1116). Another mother emphasised her son’s ‘perfectionistic streak’: ‘he doesn’t want to fail, and he doesn’t want to miss stuff out, and the anxiety involved with all of that means that he’s just got to cover everything, so he will either cover everything or not start it all’ (P1508).

Participants were also clear that sometimes other, *non-executive factors made everyday tasks even more challenging* (subtheme 2.3). These factors included motor issues ( ‘things like coordination in folding clothes, he’s not good at’; P1118), attention difficulties ( ‘when I don’t take my (ADHD) medication, it’s incredibly, incredibly hard for me to sit still’; YP1116) and comprehension issues. Indeed, in describing their challenges with following instructions, sometimes it could be about them ‘not being detailed enough’ (YP1116) or sufficiently clear: ‘sometimes, the way people explain things to me can be a little bit difficult. If it’s clear to me, then I’ll find it easy and I’ll get on with it. But sometimes I think, ‘Can you say that again please?’ (YP524). Parents noted these issues became more difficult ‘as you go further up in education, where the instructions get less and less specific’ (P1508):

Sometimes he struggles when he’s reading a question, or a teacher gives him some homework, to know what to actually do and how to do it. So, they just say, ‘research Shakespeare’. His idea of what to put in that, what’s important, what’s not important, is horrendous. (P1508)

Other parents felt, too, that understanding the intention underlying people’s instructions could be especially difficult: ‘it’s not because he’s being stubborn, but because he doesn’t understand why people are asking him to do certain things, to organise himself’ (P1118). One mother explained further: ‘the problem with planning is exacerbated by the autism, because the autism means he doesn’t always understand the meaning behind clear instructions. It’s very hard to plan if you’re doubting yourself all the time about the meaning of things’ (P1116).

### Theme 3: EF abilities – and the demands placed on them – are ever-changing

Notwithstanding these everyday challenges, both autistic young people and their mothers acknowledged that their *EF abilities have changed and continue to change over time* (subtheme 3.1). When speaking about their abilities to plan, prioritise and organise, participants felt they ‘found it hard at first but now I am okay with it’ (YP1118). They recognised they are ‘much better now than I used to be’ and hopeful that ‘the rest will work itself out perhaps as I go later into life’ (YP1506). Their mothers agreed that ‘now that he’s older, he’s really a lot better on the organisation front – much better than he used to be’ (P524) and, while ‘following instructions has always been a problem, he’s getting better with it’ (P1118). One mother described the pride she felt in witnessing her son preparing effectively for an open-book exam at college, with little support:

I keep having to remind myself that actually, this is not static. He’s come on so much. The challenge is for me to keep raising my expectations. I probably shouldn’t have been surprised, because he is improving, but that was good, and may there be many more days like that. (P1116)

Nevertheless, parents were aware that ‘adolescence is kicking in, you know, the hormones and things like that’ (P1118) and that ‘it’s obviously different now that he’s a teenager and he’s growing up, it’s different to how it used to be – he’s thinking more about adult things’ (P524). Consequently, they were concerned that *increasing expectations outpaced their (children’s) developments in EF* (subtheme 3.2) – especially since their children were ‘still requiring a huge amount of support just in structuring his work’ (P1116). One parent worried about how her child would cope in a workplace:

Now that he’s going to be 15 soon, I’m expecting him to be more independent and school are expecting him to be more independent. And I keep thinking when [child] goes into Year 10, he’s going to be doing work experience. So suddenly, it’s, ‘how’s he going to get on when he has to go and work somewhere with somebody for a week?’. (P524)

Autistic young people, too, were aware of the increasing pressures on them to be ‘more grown-up’ (P305), especially those who were beginning to transition away from highly-structured school environments: ‘in secondary school, we had a homework diary where we would keep everything in. And that’s fallen out of practice as you come into university, college or whatever, because people assume that you’re just able to maintain it yourself’ (YP1116).

Although these demands could be confronting, autistic young people nevertheless *longed for greater autonomy as they approached adulthood* (subtheme 3.3). They described how they ‘want to help’ (YP524) with chores at home, and to ‘learn how to not get distracted’ (YP1118). Some ‘value[d] the freedoms of going about my day-to-day life’ (YP1116), enjoying ‘my own space and being left alone at time when I need to be left alone’ (YP1516). Others, however, felt that they ‘can’t really plan ahead because I need to go everywhere with them [parents] because they don’t trust me by myself’ (YP1129). As one young person put it: ‘I do sometimes wish that they would leave me to make my own sort of decisions’ (YP1116).

Parents, of course, wanted their children to be more self-reliant too: ‘that’s my prayer. That they stand on their own two feet, and they don’t rely on someone, and they also become part of the community’ (P1118). They were aware of their children’s burgeoning desire for greater autonomy but also acknowledged the challenges: ‘I do probably control his life. I’m trying to back off . . .but at the same time, I’m trying not to let him have disasters’ (P1116). Other mothers, too, described the tension between supporting their children and creating opportunities for experiment: ‘I sometimes deliberately push him to arrive five minutes late, because I want him to learn to feel uncomfortable and that it’s okay, the world isn’t going to collapse’ (P1508). They also recognised that ‘as a mother, it’s just a very, very narrow line to tread’ (P1116): ‘I suppose I’ve been more gentle than I should be, because he’s right at the end of his A-Levels.^2^ He’s been under a lot of pressure, and it’s not been the time, I don’t think’ (P506).

## Discussion

We asked autistic young people and their parents directly about their or their child’s everyday experiences of executive control. Contrary to suggestions that autistic people have limited self-awareness (Frith & Happé, 1999; Lombardo et al., 2011; Zahavi, 2010), including sometimes from their own parents, autistic young people in our study provided profound insights. They told us that their EF skills were highly variable, acutely dependent on the context in which they were deployed – that is, in often uncertain, demanding and emotionally ‘hot’ circumstances – and potentially related to differences in the way that they process information more broadly. These findings might help to explain the mixed and modest effects of existing EF studies using traditional lab-based assessments (Demetriou et al., 2018). They also have important theoretical and empirical implications.

During the interviews, autistic young people spoke of their challenges with planning and, to a lesser extent, cognitive flexibility and working memory, consistent with the extant literature using lab-based tasks (Demetriou et al., 2018). In some regards, autistic young people’s observations of their own EF skills appear to map on to existing, theoretical models, which emphasise a set of core EF components – set-shifting, updating (working memory) and inhibitory control – that come together to support the execution of complex, higher-order EF skills, like problem solving, planning and navigation (Diamond, 2013; Friedman & Miyake, 2017; Miyake et al., 2000). During development, these components are thought to become progressively specialised over time, with their integration resulting in gradually more complex EF skills (Garon et al., 2008). Autistic young people’s executive challenges could therefore be the result of developmental differences in individual EF components and/or their integration. It is intriguing that our participants’ reports emphasised challenges with the more complex, higher order skills, such as planning, organising and prioritising.

There were, however, two important ways in which our interviewees’ reports did *not* map straightforwardly onto existing models. The first was that they also reported key differences in information processing – specifically, a tendency to focus attention on one thing at a time. This serial style of processing and the intensity in which it can manifest has long been described by autistic people as ‘monotropism’ (Murray et al., 2005), but has received scant attention by researchers. Given the prolonged development of EF and the degree of neural plasticity during childhood (Huttenlocher, 2002; Nelson, 1999), it is possible that monotropism interacts with EF – both in the performance of everyday tasks as well as in its development. In the neurotypical population, prominent accounts of attentional control, the ability to focus on a task and ignore irrelevant information, have suggested that developmental gains in attentional processes provide children with greater executive control over action (Posner & Rothbart, 2000) – indeed attention is considered ‘a basic building block for the EF system’ (Garon et al., 2008, p. 51). Developmental differences in the attentional networks of autistic children should therefore have substantial impact on both the expression of EF in everyday scenarios and in the emergence of (components of) EF (see Pellicano, 2012). The inter-relationship between aspects of attention (even as traditionally described; Posner & Rothbart, 2000) and components of EF has hitherto been unaddressed in autistic people. Future research should investigate how monotropism overlaps with the central attention system (Posner & Rothbart, 2000), and its interactions with EF, in both its expression and its emergence over developmental time.

The second way in which standard theoretical models of EF did not map on to our interviewees’ insights relates to the deployment of everyday EF. Autistic young people and their mothers repeatedly spoke of how their executive skills were sometimes enabled, and at other times limited, by the context in which they were doing the task, their level of anxiety, the clarity of the task instructions and their motivation/interest in the task. For example, difficulties understanding task instructions could lead participants to feel uncertain about whether they were completing a goal as intended (by others), and this uncertainty could cause heightened anxiety and sometimes inertia (see Buckle et al., 2021; Rapaport et al., 2023). Autistic young people’s motivation towards the task also mattered enormously in executing everyday tasks (Marks et al., 2000). While the influence of these factors in the expression of EF may seem intuitive, they are almost wholly unaccounted for in traditional lab-based EF assessments, which are intentionally designed to be devoid of context. Nor are they discussed in models of autistic EF (Hughes et al., 1994; Rajendran & Mitchell, 2007). One way to address this omission is to investigate systematically the ways in which these factors affect autistic young people’s performance on traditional, lab-based EF tasks, by experimentally manipulating, for example, the extent to which the task is motivating or anxiety-inducing.

While such investigations might go some way towards understanding the inconsistencies within the EF literature, they do not address the fact that the decontextualised nature of existing, lab-based EF tasks fails to capture the dynamic and situated nature of everyday EF. There have been some encouraging attempts to tackle the issue of ecological validity in autistic EF research, including using virtual reality (e.g. Rajendran et al., 2011) and real-life measures (Kenny et al., 2023; Kenworthy et al., 2020; Mackinlay et al., 2006) that directly address concerns about the representativeness of (executive) task demands. One such study used a naturalistic EF task in which autistic adolescents were asked to prepare food, drinks and materials for an event – and thus assessed the ability to prioritise, generate a plan, chain multiple goals together, and execute a plan in the context of sensory or anxiety-provoking conditions (Kenny et al., 2023). Autistic adolescents under-performed, on average, relative to non-autistic adolescents, specifically producing lower quality products (e.g. poorly assembled sandwich) and using less efficient strategies – in ways that are highly similar to parents’ reports here.

Kenworthy et al. (2020) have also designed a standardised, objective measure, the Executive Function Challenge Task (EFCT), which assesses flexibility and planning within the context of provocative social scenarios. A study with autistic young people (*n* = 129) demonstrated its promise as a test of everyday EF. The EFCT showed good psychometric properties and its scores were significantly correlated with parent-reported EF (Kenworthy et al., 2020). The strength of both everyday executively-demanding tasks (Kenny et al., 2023; Kenworthy et al., 2020) is that they seek to replicate real-world EF demands, which are often social and emotionally-charged (‘hot’) in nature. Nevertheless, it remains challenging to disentangle the roles of executive and non-executive factors on task performance and isolate the specific components of EF that may be particularly challenged.

Solving this problem – that is, designing EF tasks that meet the thresholds for representativeness and generalisability and that also permit the isolation of specific mechanisms – may well require a thoroughgoing rethink of the way in which we approach the study of autistic cognition. One potentially promising approach is to integrate subjective, first-person experiences of EF – like the ones reported herein – with objective, third-person behaviour (Jack & Roepstorff, 2002; Kingstone et al., 2008). Having now elicited autistic young people’s phenomenological experiences of EF (step 1), the next step would be to conduct meticulous observations and/or measurement of their executive skills ‘in the wild’ (e.g. carrying out an errand) (step 2). Such measurement would be followed in turn by detailed retrospective interviews with participants to provide additional evidence about the specific task demands and the strategies used during task performance, and to draw attention to the person’s experience (i.e. of ‘what it is like’ to do the task) (step 3) (Jack & Roepstorff, 2002). We would then use this information to test against standard lab-based EF measures, and design and implement ecologically-sensitive experiments (in situ and/or in virtual reality) targeting the processes of interest determined from steps 1 and 2.

Adopting this fine-grained and multi-layered approach is especially important because the qualitative responses reported herein clearly showed that autistic young people’s EF is not always experienced as a difficulty or problem; it becomes so predominantly when demand exceeds capacity. Our participants – both young people and their parents – highlighted how the increasing demands placed on them appeared to outpace developments in EF, resulting in EF difficulties becoming more apparent as they approach adulthood (Cribb et al., 2019). This finding is consistent both with cross-sectional studies demonstrating that autistic participants’ EF difficulties become more pronounced from childhood into adolescence using teacher- (Kouklari et al., 2018) and parent-reported EF (Rosenthal et al., 2013) and with longitudinal studies showing that, as autistic participants get older, their standardised scores on measures of adaptive behaviour markedly decline (Gotham et al., 2012; Pellicano et al., 2020; Pugliese et al., 2016; Salomone et al., 2018). Understanding both the demands of the everyday environments in which autistic people live and how these might be aligned – or misaligned – with individual (EF) characteristics is critical for understanding the conditions under which EF might, and where to target the most appropriate support (Lai et al., 2020; Mandy, 2022).

Regarding support, although there was remarkable convergence between young people’s and parents’ reports generally, one clear point of tension concerned the desire for greater autonomy. Parents were acutely aware they often provided external sources of executive control *for* their child, akin to a ‘conductor’ – managing, directing, organising, and planning – especially within the context of the demands of adolescence. Yet, they were also concerned they were not providing their children with sufficient opportunities to exercise and develop these skills. Young people themselves, like those in previous studies, worried about their ability to cope with the increasing demands that lie ahead (Browning et al., 2009; Cribb et al., 2019). Addressing this issue is clearly not straightforward but collaborating actively with individual young people (Pellicano & Stears, 2011) would appear to be a fundamental first step towards resolving the observed tensions.

### Limitations

This study has several limitations. First, our interview schedule was informed by existing models of EF (e.g. Pellicano, 2012), which meant we asked directly about issues with autistic young people’s planning, cognitive flexibility and working memory skills. Given that these models are derived from normative conceptualisations of EF, adopting this approach may have constrained our attempts to understand everyday EF skills in our autistic participants. Notwithstanding, both the semi-structured nature of our interviews and our inductive analytic approach allowed us to identify aspects of autistic cognition that often remain unexamined.

Second, our participants were autistic young people without an intellectual disability, who could participate in a semi-structured interview, meaning our findings cannot address the executive skills of those with intellectual disabilities and/or who are non-speaking. That said, it is likely that similar factors (context, anxiety, motivation, language) are likely to moderate the deployment of EF in everyday life just as they did for our participants, although future research will need to examine this issue directly.

Third, we included autistic young people from a reasonably wide age range (12–19 years). Adolescence is a time during which there is a boost in growth of executive and other skills (Blakemore & Choudhury, 2006; Diamond, 2014; Uddin, 2021), including in autistic adolescents (see Demetriou et al., 2018). Although our interviewees noted how their own or their children’s executive skills seemed to have changed over time, the qualitative nature of our study could not delineate any specific age-related changes in EF. Future research should seek to examine any such changes, particularly during significant periods of transition (e.g. into and out of secondary school), when external demands on EF might be heightened.

Fourth, a significant minority of our sample of young people were reported to have co-occurring neurodevelopmental conditions, including ADHD, which may have made it difficult to understand the phenomenological experience of EF for autistic young people specifically. ADHD young people also have often-substantial EF difficulties, so it will be important to delineate the ways in which their everyday EF experiences are distinct. Given that autistic young people are as much as 20 times more likely than their non-autistic peers to have attentional difficulties (Ghirardi et al., 2018), our inclusion of autistic young people with co-occurring conditions is in keeping with our commitment to understand the lived realities of EF challenges.

Finally, we did not invite autistic young people to help to make decisions about the design and conduct of this study or the interpretation of findings. Future research should ensure such involvement so that the research is more thoroughly attentive to autistic young people’s needs, preferences and interests.

## Conclusion

Our current laboratory-based, researcher-designed measures of EF do not capture the contextualised nature of everyday EF, or the important role of other factors that might influence the deployment and development of executive control. The insights of the autistic young people and their parents could help redress the mismatch between lab and life – and lead to a conceptualisation of EF that maps more accurately captures these experiences and, as a result, better serves their needs.

## Supplemental Material

sj-doc-1-aut-10.1177_13623613231224093 – Supplemental material for Everyday executive function issues from the perspectives of autistic adolescents and their parents: Theoretical and empirical implications Supplemental material, sj-doc-1-aut-10.1177_13623613231224093 for Everyday executive function issues from the perspectives of autistic adolescents and their parents: Theoretical and empirical implications by Lorcan Kenny, Anna Remington and Elizabeth Pellicano in Autism

## Notes

1. lastminute.com is a UK-based online travel and leisure retailer.

2. A-Levels (Advanced Level qualifications) are a UK subject-based, school-leaving qualification, usually studied over two years, for students aged 16 and above.
