# Supplementary material for: Everyday executive function issues from the perspectives of autistic adolescents and their parents: Theoretical and empirical implications
Source: Autism. 2024 Jan 19;28(9):2204–17. doi: 10.1177/13623613231224093 (PMC11408970; doi:10.1177/13623613231224093)
Supplement: sj-doc-1-aut-10.1177_13623613231224093 – Supplemental material for Everyday executive function issues from the perspectives of autistic adolescents and their parents: Theoretical and empirical implications [file sj-doc-1-aut-10.1177_13623613231224093.doc]

**Interview questions with parents**

*We have 6 main questions about (1) how your child is doing in general, (2) how they are at managing their time, (3) how they find planning ahead, (4) remembering to do things, (5) how they are at multi-tasking and finally (6) whether you think being on the autism spectrum effects any of these things. You can let me know if you don’t want to talk about any of these or you can ask me to skip any questions you like.*

1. **To start, would you mind telling me a little about how your son/daughter has been getting on lately?**

Probe questions:

- How have they been finding schoolwork?
- At school, what things do they like/are they good at?
  - Why do you think they find those things easy?
- What things do they find more difficult?
  - Why do you think they find those things difficult?
- How have things been going for them outside of school, say with friends or at home?
- What do you think they find difficult about getting on with friends family?
  - Why do you think this might be?
- What about keeping organised at home?
  - Are they the type of person who keeps their room neat and tidy?
  - Do they ever have to manage their own money?

1. **Managing their time**
   1. Can you tell me about a time when managing their time didn’t go so well?
      1. Why do you think it didn’t go well?
      2. What could they have done better in that situation?
   2. Can you tell me about a time when they were very good at managing their time and it went well?
      1. Why do you think it went well?

Additional probe questions:

- - Do they tend to get all their homework done on time?
  - Do you have enough time to get all their work done in school?
  - How do they find managing their time between schoolwork and other activities?
  - Do they get chores to do at home?
    - Do you think they find it easy to get them all done within the time they are given?
  - Do they find it easy to leave on time before they go to school or somewhere else?
  - Are they good at fitting in all their plans in the evenings and weekends?
  - Do they generally manage to get all of their homework done on time?
  - Do you think they are a punctual person?

1. **How do you think your son/daughter is at planning ahead of time for things?**
   1. Can you give me an example of when they planned something and it went really well?
      1. Why do you think it didn’t go well?
      2. What could they have done better in that situation?
   2. Can you give me an example of when they planned something and it didn’t go well?
      1. Why do you think it went well?

Additional probe questions:

- - Do you think they are good at coming up with a plan when they need to?
  - Do they tend to finish things they begin?
    - Why is that?
  - Do they plan or manage their own money at all?
    - Do you think they have they ever found this difficult?
  - Do they plan ahead of time what to do in school holidays?
    - How do they find that?
  - How do they feel when somebody changes a plan at the last minute?
  - How good are they at adjusting to the new plan?
  - Can you give me an example of when a plan changed for them?
    - Tell me what happened, how they reacted, what was the outcome?
  - How do you think they are at planning over a smaller amount of time?
    - For example, planning a route around a shopping centre?
    - Or, planning all the jobs they need to do before leaving the house?

1. **Are they good at remembering things people tell them?**
   1. **Can you tell me about a time when this went really well for them?**
      1. Why do you think it didn’t go well?
      2. What could they have done better in that situation?
   2. **Can you tell me about a time when this didn’t go so well for them?**
      1. Why do you think it went well?

Probe questions:

- - Are they good at remembering to do something?
    - For example, getting somebody a birthday card, stopping by the shop on their way somewhere or calling/texting somebody to arrange a plan.
  - How do they find it when a teacher/parent gives them instructions, are they able to remember them all?
  - Do you know if they use any tricks to help with this?
    - Things like setting alarms or writing notes for themselves?
    - Do you think they help?
  - How about when they go into a shop, do you know if they find it easy to remember everything they went in for?
  - Do they often forget to bring things to school that they need?
  - Have they ever forgotten to do homework or done the wrong things for homework?
  - Are they good at remembering phone numbers or directions people give you?

1. **We often have to concentrate on doing many things at the same time, how do you think your son/daughter is at doing more than one thing at a time?**
   1. **Can you tell me about a time that they found it hard to multitask and it didn’t go so well for them?**
      1. Why do you think it didn’t go well?
      2. What could they have done better in that situation?
   2. **Can you tell me about a time that they found it easy to multitask and it did go well for them?**
      1. Why do you think it went well?

Probe questions:

- - What about listening to somebody while looking at their phone/watching television?
  - Do they ever cook/bake?
    - If so, do they find it stressful?
    - If so, why?
  - Do they ever do more than one thing at a time when you probably should not?
    - For example, going on your phone in class.
  - Are they good at switching between activities to get everything done on time?
    - For example, moving from homework on one subject to another.
  - Do they play any sports or have hobbies that require them to do many things at once?
    - How do they find them?
- How easy do they find it to switch between doing something they like and something they don’t like doing?
  - Switching from watching TV to doing chores for example.
- Are there things they find hard not to do, even if they know they shouldn’t do them?
  - For example, spending money when they need to save it or watching tv when they have homework to do.

1. **Do you think that being on the autism spectrum effects how they have been getting on with these types of things?**

Probe questions:

- Do you think the things they struggle with are the same or different from others their age?
- What about the things tey are good at, are they similar or different from others their age, do you think?
- Do you think being autistic explains any part of their organisational or planning skills?
- Some people say that being autistic makes it harder to switch between one activity and other. Would you agree?
- Others say that having an autism diagnosis makes it hard to do more than one thing at a time, do you think that is true for your son/daughter?
- Would you like it if they got more help with these types of skills?
- If there was something they could do to improve their planning and organising skills would you do it?
- Would you have any worries about trying to change these skills?
- Is there anything you would like to add that I haven’t asked about?

**Interview questions with young person**

*We have 6 main questions about (1) how you are in general, (2) how you manage your time, (3) planning ahead, (4) remembering to do things, (5) how you are at multi-tasking and finally (6) whether you think being on the autism spectrum effects any of these things. You can let me know if you don’t want to talk about any of these or you can ask me to skip any questions you like.*

1. **To start, would you mind telling me a little about how you have been getting on lately?**

Probe questions:

- How have you been finding schoolwork?
- At school, what things do you like/are you good at?
  - Why do you think you find those things easy?
- What things do you find more difficult?
  - Why do you think you find those things difficult?
- How have things been going outside of school, say with friends or at home?
- What do you find difficult about getting on with friends family?
  - Why do you think this might be?
- What about keeping organised at home?
  - Are you the type of person who keeps your room neat and tidy?
  - Do you ever have to manage your own money?

1. **Managing your time**
   1. Can you tell me about a time when managing your time didn’t go so well?
      1. Why do you think it didn’t go well?
      2. What could you have done better in that situation?
   2. Can you tell me about a time when you were very good at managing your time and it went well?
      1. Why do you think it went well?

Additional probe questions:

- - Do you get all your homework done on time?
  - Do you have enough time to get all your work done in school?
  - How do you find managing your time between schoolwork and other activities?
  - Do you get chores to do at home?
    - Do you find it easy to get them all done within the time you are given?
  - Do you find it easy to leave on time before you go to school somewhere else?
  - Are you good at fitting in all your plans in the evenings and weekends?
  - Do you generally manage to get all of your homework done on time?
  - Do you think you are a punctual person?

1. **How do you think you are at planning ahead of time for things?**
   1. Can you give me an example of when you planned something and it went really well?
      1. Why do you think it didn’t go well?
      2. What could you have done better in that situation?
   2. Can you give me an example of when you planned something and it didn’t go well?
      1. Why do you think it went well?

Additional probe questions:

- - Do you think you are good at coming up with a plan when you need to?
  - Do you tend to finish things you begin?
    - Why is that?
  - Do you plan or manage your own money at all?
    - Have you ever found this difficult?
  - Do you plan ahead of time what to do in school holidays?
    - How do you find that?
  - How do you feel when somebody changes a plan at the last minute?
  - How good are you at adjusting to the new plan?
  - Can you give me an example of when a plan changed for you?
    - Tell me what happened, how you felt, what was the outcome?
  - How do you think you are at planning over a smaller amount of time?
    - For example, planning a route around a shopping centre?
    - Or, planning all the jobs you need to do before leaving the house?

1. **Are you good at remembering things people tell you?**
   1. **Can you tell me about a time when this went really well for you?**
      1. Why do you think it didn’t go well?
      2. What could you have done better in that situation?
   2. **Can you tell me about a time when this didn’t go so well for you?**
      1. Why do you think it went well?

Probe questions:

- - Are you good at remembering to do something?
    - For example, getting somebody a birthday card, stopping by the shop on your way somewhere or calling/texting somebody to arrange a plan.
  - How do you find it when a teacher/parent gives you instructions, are you able to remember them all?
  - Do you use any tricks to help with this?
    - Do you set alarms or write notes for yourself?
    - Do they help?
  - How about when you go into a shop, do you find it easy to remember everything you went in for?
  - Do you often forget to bring things to school that you need?
  - Have you ever forgotten to do homework or done the wrong things for homework?
  - Are you good at remembering phone numbers or directions people give you?

1. **We often have to concentrate on doing many things at the same time, how do you think you are at doing more than one thing at a time?**
   1. **Can you tell me about a time that you found it hard to multitask and it didn’t go so well for you?**
      1. Why do you think it didn’t go well?
      2. What could you have done better in that situation?
   2. **Can you tell me about a time that you found it easy to multitask and it did go so well for you?**
      1. Why do you think it went well?

Probe questions:

- - What about listening to somebody while looking at your phone/watching television?
  - Do you ever cook/bake?
    - If so, do you find it stressful?
    - If so, why?
  - Do you ever do more than one thing at a time when you probably should not?
    - For example, going on your phone in class.
  - Are you good at switching between activities to get everything done on time?
    - For example, moving from homework on one subject to another.
  - Do you play any sports or have hobbies that require you to do many things at once?
    - Tell me, how do you find them?
- How easy do you find it to switch between doing something you like and something you don’t like doing?
  - Switching from watching TV to doing chores for example.
- Are there things you find hard not to do, even if you know you should do them?
  - For example, spending money when you need to save it or watching tv when you have homework to do.

1. **Do you think that being on the autism spectrum effects how you have been getting on with these types of things?**

Probe questions:

- Do you think the things you struggle with are the same or different from others your age?
- What about the things you are good at, are they similar or different from others your age, do you think?
- Do you think being autistic explains any part of your organisational or planning skills?
- Some people say that being autistic makes it harder to switch between one activity and other. Would you agree?
- Others say that having an autism diagnosis makes it hard to do more than one thing at a time, do you think that is true for you?
- Would you like it if you got more help with these types of skills?
- If there was something you could do to improve your planning and organising skills would you do it?
- Would you have any worries about trying to change these skills?
- Is there anything you would like to add that I haven’t asked about?
